# Incidence rates of tuberculosis and inflammatory bowel disease in patients with ankylosing spondylitis treated with biologics in Korea

**DOI:** 10.1093/rheumatology/keaf038

**Published:** 2025-01-24

**Authors:** Oh Chan Kwon, Hye Sun Lee, Juyeon Yang, Thomas Paul, Hyerim Jin, Youkyung Lee, Min-Chan Park

**Affiliations:** Division of Rheumatology, Department of Internal Medicine, Yonsei University College of Medicine, Seoul, South Korea; Biostatistics Collaboration Unit, Yonsei University College of Medicine, Seoul, South Korea; Biostatistics Collaboration Unit, Yonsei University College of Medicine, Seoul, South Korea; Novartis Korea Ltd, Seoul, South Korea; Novartis Korea Ltd, Seoul, South Korea; Novartis Korea Ltd, Seoul, South Korea; Division of Rheumatology, Department of Internal Medicine, Yonsei University College of Medicine, Seoul, South Korea

**Keywords:** ankylosing spondylitis, tumour necrosis factor inhibitor, interleukin-17 inhibitor, tuberculosis, inflammatory bowel disease

## Abstract

**Objective:**

To describe the incidence rates of inflammatory bowel disease (IBD) and tuberculosis (TB) in Korean patients with ankylosing spondylitis receiving biologics.

**Methods:**

Data from a Korean claims database between 2010 and 2021 was used to calculate crude incidence rates of TB and IBD using number of events and total patient-years (PYs).

**Results:**

Overall, 43 643 and 43 396 patients were included in TB and IBD cohorts, respectively. Exposure-adjusted incidence rates (EAIRs) of TB for non-exposure, TNF inhibitors (TNFis), and IL-17 inhibitors (IL-17is) were 0.14, 0.25 and 0.12 and of IBD were 0.18, 0.19 and 0.44 per 100 PYs, respectively. Incidence rates during biologic DMARD (bDMARD) non-exposure, adalimumab, etanercept, golimumab, infliximab, secukinumab and ixekizumab exposures for TB were 13.96, 27.79, 14.28, 21.19, 33.62, 12.74 and 0.00 and for IBD were 18.29, 19.98, 22.41, 18.85, 15.73, 44.99 and 0.00 per 10 000 PYs, respectively. Compared with bDMARD non-exposure, adalimumab, golimumab and infliximab exposures were associated with a significantly higher risk of TB. Etanercept and secukinumab exposure showed no significant increase in risk of TB. Compared with bDMARD non-exposure, exposure to biologics did not show a significant difference in risk of IBD.

**Conclusion:**

EAIRs of TB and IBD with use of IL-17is in patients with AS were within anticipated low range. IL-17is had numerically lower incidence of TB, and numerically higher incidence of IBD compared with TNFis. Notably, secukinumab showed no increased risk of TB compared with bDMARD non-exposure. Neither TNFis nor IL-17is showed increased risk of IBD compared with bDMARD non-exposure.

Rheumatology key messages

- Exposure-adjusted incidence rates of TB and IBD with IL-17is in patients with ankylosing spondylitis were within anticipated low range.
- Secukinumab was not associated with the risk of incident TB compared with bDMARD non-exposure.
- Neither TNFis nor IL-17is showed an increased risk of IBD when compared with bDMARD non-exposure.

## Introduction

Ankylosing spondylitis (AS) is a chronic immune-mediated rheumatic disease characterized by inflammation and formation of new bone, predominantly affecting the axial skeleton and sacroiliac joints [1, 2]. It has a profound impact on patients’ health-related quality of life [3, 4] and is associated with several comorbidities such as cardiovascular disease, dyslipidaemia, osteoporosis, malignancies, pulmonary disease and depression [5].

Use of NSAIDs is recommended as the first-line treatment in AS. Biologic DMARDs (bDMARDs) including TNF inhibitors (TNFis) and IL-17 inhibitors (IL-17is) are recommended when treatment with NSAIDs is inadequate [6–8]. bDMARDs are associated with adverse events (AEs) that are linked to their unique mechanism of action [9]. Moreover, these drugs have the potential to be responsible for severe AEs and uncommon, unpredictable AEs that are challenging to identify during pre-marketing clinical trials [10]. Post-marketing safety profiles of bDMARDs has been well-evaluated using retrospective data from the registries [11, 12].

Tuberculosis (TB) is an opportunistic infection occurring in patients with autoimmune rheumatic diseases, such as AS. The risk of latent TB reactivation or *de novo* TB cases is increased in these patients treated with biologic agents especially TNFis [13, 14]. This increased risk is due to cell-mediated immune defects associated with inflammation and the impact of TNFis on the structure of TB-related granulomas. TNF-α plays an important role in the formation of granulomas that are crucial for containing bacterial spread in the body. TNF-α is essential for activating macrophages and recruiting immune cells to the granuloma. Granulomas dissolve in the absence of TNF-α, allowing re-growth and spread of mycobacteria [15, 16]. Compared with TNFis, the mechanism of action of IL-17is is more targeted, resulting in fewer warnings and precautions in general. In patients who are at a high risk for TB and hepatitis B virus infection, IL-17is have been proven to be safer [17].

Korea has the second highest incidence of TB (39 per 100 000 population) among member countries of the Organization for Economic Co-operation and Development [18]. A study from South Korea reported a high risk of TB in patients with AS on TNFi therapy, with an incidence rate of 600.2 per 100 000 patient-years (PYs) [19].

Inflammatory bowel disease (IBD) is one of the common extra-articular manifestations of AS [20]. Studies have reported that patients with AS have a higher occurrence and prevalence of IBD compared with the general population [21, 22].

For a long time, TNFis were the only biologic agents available for the treatment of AS in patients who fail NSAIDs. The emergence of IL-17is and Janus kinase inhibitor (JAKi) provides additional treatment options to these patients. As might be expected, the biologics that are approved recently have limited safety data, and there is a paucity of information with respect to the long-term safety of biologics in AS apart from TNFis [6]. The growing use of non-TNFi agents highlights the need for more real-life studies that would compare the safety of TNFis and other biologics used in these patients.

In Korean patients with AS, over the past decade significant clinical experience has been gained in the use of biologic agents including secukinumab, a fully human anti-IL-17A monoclonal antibody. It is imperative to have a comprehensive understanding of the current status of biologic agents, which includes assessing the long-term real-world safety outcomes and monitoring the safety profile of special interest (SPSI), especially IBD and TB. Using data from a national claims database, the present study aimed to describe the incidence rates of IBD and TB in patients with AS receiving biologic agents (TNFis and IL-17is) during the study period.

## Methods

### Data source and study cohort

This retrospective nationwide cohort study used data acquired from the national health service agency and Health Insurance Review and Assessment (HIRA) Service database, a Korean nationwide registry that includes information on demographics, disease diagnoses and medical treatments, and covers ∼97% of South Korea’s population [23]. Patients diagnosed with AS and treated with biologic agents between 2010 and 2021 were selected from the database. The follow-up period of the study cohort was from the diagnosis of AS to December 2021 or the date of occurrence of TB or IBD, whichever occurred first.

### Outcomes and covariates

TB was defined as the International Classification of Diseases (ICD)-10 codes A15–19 with prescription for at least two of the first-line drugs for TB (isoniazid, rifampin/rifampicin, pyrazinamide, ethambutol) [24]. IBD was defined as ICD-10 codes K50 and K51 [25].

The primary objective was to evaluate the exposure-adjusted incidence rates (EAIRs) of SPSI for TNFis and IL-17is and the incidence rates of SPSI for each individual biologic agent during the study period in patients with AS from the HIRA database. The secondary objective was to comprehensively assess and characterize the risk associated with the use of TNFis and IL-17is within distinct subgroups stratified by age, sex and comorbidities.

Comorbidities, such as hypertension, type 2 diabetes, dyslipidaemia, chronic kidney disease (CKD), psoriasis and uveitis, were included as covariates. The definitions of the comorbidities are as follows: hypertension: ICD-10 codes I10–I13 and I15 with prescriptions for antihypertensive agents; type 2 diabetes: ICD-10 codes E11–14 and at least one annual claim of a prescription of anti-diabetic agents; dyslipidaemia: ICD-10 code E78 with prescriptions for lipid-lowering agents; and CKD: ICD-10 code N18 or N19 [24, 26, 27]. The following medications used during follow-up were assessed as covariates: methotrexate, sulfasalazine, glucocorticoid, non-selective NSAIDs and selective cyclooxygenase-2 inhibitors.

### Statistical analyses

Patient characteristics are presented as means (s.d.) and number (%) for continuous and categorical variables, respectively. The crude incidence rates during exposure to TNFis, IL-17is, or none were calculated using the number of events and total PYs for the primary outcomes. Time-dependent Cox regression models were used. Hazard ratios (HRs) and 95% CI for incident primary outcomes were estimated comparing those who were exposed to TNFis *vs* non-exposed, those who were exposed to IL-17is *vs* non-exposed, and those who were exposed to IL-17is *vs* TNFis. Also, TNFis were further categorized into receptor fusion protein (etanercept) and monoclonal antibodies (adalimumab, golimumab and infliximab). HRs and 95% CI for incident primary outcomes were additionally calculated comparing those who were exposed to receptor fusion protein *vs* non-exposed, those who were exposed to monoclonal antibodies *vs* non-exposed, and those who were exposed to monoclonal antibodies *vs* receptor fusion protein. Univariable models were performed followed by multivariable models adjusted for age, sex, comorbidities and medications. A *P*-value <0.05 was considered as significant. Analyses were conducted using SAS version 9.4 (SAS Institute, Cary, NC, USA) and R version 4.3.2 (R Foundation for Statistical Computing, Vienna, Austria).

### Ethics approval

This study was conducted in accordance with the principles embodied in the Declaration of Helsinki and was approved by the Institutional Review Board (IRB) of the Gangnam Severance Hospital (IRB approval no.: 3-2022-0159), which waived the requirement for the acquisition of informed consent from patients owing to the retrospective nature of this study.

## Results

A total of 71 001 patients with AS were identified. Among these patients, those who (i) were diagnosed with AS prior to 2010 (*n* = 25 943), (ii) were exposed to medication prior to AS diagnosis (*n* = 758), (iii) had a history of TB (*n* = 55) or IBD (*n* = 69) prior to AS diagnosis, and (iv) developed TB (*n* = 602) or IBD (*n* = 835) within 3 months after being diagnosed with AS were subsequently excluded. The remaining 43 643 and 43 396 patients comprised the TB and IBD study cohort, respectively (Supplementary Figs S1 and S2, available at *Rheumatology* online).

### Patient characteristics and incidence rate of TB

Of the 43 643 patients (mean age: 41.4 [16.4] years; male: 70.7%) included in the TB study cohort, 43 643, 5674, 2614, 2670, 2150, 430 and 31 patients contributed PYs to bDMARD non-exposure, adalimumab exposure, etanercept exposure, golimumab exposure, infliximab exposure, secukinumab exposure and ixekizumab exposure, respectively (Supplementary Fig. S1, available at *Rheumatology* online). Table 1 presents the prevalence of comorbidities as well as the medications used during the study period. Uveitis (28.7%) was the most common comorbidity and non-selective NSAIDs (91.3%) the most common concomitant medication. The EAIRs of TB for non-exposure, TNFis and IL-17is were 0.14, 0.25 and 0.12 per 100 PYs, respectively (Table 2).

**Table 1.** Characteristics of the TB and IBD study population

	TB (*n* = 43 643)	IBD (*n* = 43 396)
Demographics		
Age, mean (s.d.), years	41.4 (16.4)	41.6 (16.5)
Male, *n* (%)	30 839 (70.7)	30 694 (70.7)
Comorbidities		
Hypertension, *n* (%)	9061 (20.8)	9098 (21.0)
Type 2 diabetes, *n* (%)	3289 (7.5)	3339 (7.7)
Hyperlipidaemia, *n* (%)	7113 (16.3)	7101 (16.4)
CKD, *n* (%)	664 (1.5)	664 (1.5)
IBD, *n* (%)	1302 (3.0)	—
Psoriasis, *n* (%)	2661 (6.1)	2640 (6.1)
Uveitis, *n* (%)	12 504 (28.7)	12 406 (28.6)
Medications		
Methotrexate, *n* (%)	6820 (15.6)	6766 (15.6)
Sulfasalazine, *n* (%)	25 434 (58.3)	25 216 (58.1)
Glucocorticoids, *n* (%)	36 159 (82.9)	35 939 (82.8)
Nonselective NSAIDs, *n* (%)	39 816 (91.3)	39 675 (91.4)
Selective COX-2 inhibitors, *n* (%)	22 538 (51.6)	22 347 (51.5)

CKD: chronic kidney disease; COX-2: cyclooxygenase-2; IBD: inflammatory bowel disease; TB: tuberculosis.

**Table 2.** EAIRs/100 patient-years for TB and IBD

	Event		Total patient-years		EAIRs/100 patient-years	
	TB	IBD	TB	IBD	TB	IBD
Non-exposure	251	325	179 768.2	177 686.4	0.14	0.18
TNFis	120	94	47 857.4	48 257.2	0.25	0.19
IL-17is	1	3	803.8	686.9	0.12	0.44

EAIR: exposure-adjusted incidence rate; IL-17is: IL-17 inhibitors; TNFis: TNF inhibitors.

The incidence rates of TB during bDMARD non-exposure, adalimumab exposure, etanercept exposure, golimumab exposure, infliximab exposure, secukinumab exposure and ixekizumab exposure were 13.96, 27.79, 14.28, 21.19, 33.62, 12.74 and 0.00 per 10 000 PYs, respectively (Table 3). As the PYs of ixekizumab exposure was too short and no events occurred during this short exposure duration, ixekizumab exposure was excluded in the Cox models.

**Table 3.** Incidence rates of TB

	Events	Patient-years	IR/10 000 patient-years (95% CI)
Non-exposure	251	179 768.2	13.96 (12.24, 15.69)
Adalimumab	57	20 512.4	27.79 (20.58, 34.99)
Etanercept	14	9802.9	14.28 (6.81, 21.76)
Golimumab	17	8024.3	21.19 (11.13, 31.25)
Infliximab	32	9517.9	33.62 (21.99, 45.25)
Secukinumab	1	784.7	12.74 (−12.22, 37.71)
Ixekizumab	0	19.1	0.00 (0.00, 0.00)

IR: incidence rate; TB: tuberculosis.

### Risk of incident TB across different bDMARDs

Table 4 presents the HRs for incident TB according to bDMARD exposure. Compared with bDMARD non-exposure, adalimumab exposure (adjusted HR [aHR], 2.174; 95% CI: 1.613, 2.930; *P* < 0.0001), golimumab exposure (aHR, 2.296; 95% CI: 1.391, 3.788; *P* = 0.0011) and infliximab exposure (aHR, 2.403; 95% CI: 1.647, 3.506; *P* < 0.0001) were associated with a significantly higher risk of TB. On the other hand, etanercept exposure (aHR, 1.007; 95% CI: 0.585, 1.733) and secukinumab exposure (aHR, 1.531; 95% CI: 0.212, 11.039) showed no significant increase in the risk of TB, compared with bDMARD non-exposure.

**Table 4.** Risk of incident TB according to bDMARD exposure

	Univariable model		Multivariable model	
	Unadjusted HR (95% CI)	*P*	Adjusted HR^a^ (95% CI)	*P*
bDMARD non-exposure *vs*				
Adalimumab	2.097 (1.573, 2.797)	<0.0001	2.174 (1.613, 2.930)	<0.0001
Etanercept	1.040 (0.607, 1.782)	0.8857	1.007 (0.585, 1.733)	0.9798
Golimumab	2.189 (1.331, 3.600)	0.002	2.296 (1.391, 3.788)	0.0011
Infliximab	2.392 (1.656, 3.457)	<0.0001	2.403 (1.647, 3.506)	<0.0001
Secukinumab	1.830 (0.255, 13.143)	0.5481	1.531 (0.212, 11.039)	0.6728
Etanercept *vs*				
Adalimumab	1.985 (1.106, 3.563)	0.0216	2.168 (1.205, 3.900)	0.0098
Golimumab	1.905 (0.928, 3.912)	0.0791	2.134 (1.037, 4.392)	0.0396
Infliximab	2.264 (1.208, 4.245)	0.0108	2.430 (1.293, 4.568)	0.0058
Secukinumab	1.726 (0.223, 13.336)	0.601	1.477 (0.19, 11.469)	0.7092

a Adjusted for age, sex, hypertension, type 2 diabetes, hyperlipidaemia, CKD, IBD, psoriasis, uveitis, methotrexate, sulfasalazine, glucocorticoids, non-selective NSAIDs and selective cyclooxygenase-2 inhibitors. bDMARD: biologic DMARD; CKD: chronic kidney disease; IBD: inflammatory bowel disease; HR: hazard ratio; TB: tuberculosis.

When compared with etanercept exposure, adalimumab exposure (aHR, 2.168; 95% CI: 1.205, 3.900; *P* = 0.0098), golimumab exposure (aHR, 2.134; 95% CI: 1.037, 4.392; *P* = 0.0396) and infliximab exposure (aHR, 2.430; 95% CI: 1.293, 4.568; *P* = 0.0058) were associated with a significantly higher risk of TB. Secukinumab exposure (aHR, 1.477; 95% CI: 0.190, 11.469) was not associated with a higher risk of TB compared with etanercept exposure.

In the exploratory subgroup analyses, similar results were observed across all subgroups except for subgroups stratified by CKD. The association between golimumab exposure (*vs* bDMARD non-exposure) and higher risk of TB was more robust in patients with CKD than in those without CKD (*P* for interaction = 0.007) (Supplementary Fig. S3, available at *Rheumatology* online).

### Patient characteristics and incidence rate of IBD

Of the 43 396 patients (mean age: 41.6 [16.5] years; male: 70.7%) included in the IBD study cohort, 43 396, 6110, 2990, 3063, 2313, 433 and 41 patients contributed PYs to bDMARD non-exposure, adalimumab exposure, etanercept exposure, golimumab exposure, infliximab exposure, secukinumab exposure and ixekizumab exposure, respectively (Supplementary Fig. S2, available at *Rheumatology* online). Table 1 presents the prevalence of comorbidities as well as the medications used during the study period. Uveitis (28.6%) was the most common comorbidity, and non-selective NSAIDs (91.4%) comprised the most common concomitant medication. The EAIRs of IBD for non-exposure, TNFis and IL-17is were 0.18, 0.19 and 0.44 per 100 PYs, respectively (Table 2).

The incidence rates of IBD during bDMARD non-exposure, adalimumab exposure, etanercept exposure, golimumab exposure, infliximab exposure, secukinumab exposure and ixekizumab exposure were 18.29, 19.98, 22.41, 18.85, 15.73, 44.99 and 0.00 per 10 000 PYs, respectively (Table 5). The incidence rates of Crohn’s disease (CD) during bDMARD non-exposure, adalimumab exposure, etanercept exposure, golimumab exposure, infliximab exposure, secukinumab exposure and ixekizumab exposure were 6.00, 10.16, 9.14, 8.81, 6.68, 29.85 and 0.00 per 10 000 PYs, respectively (Table 5). The incidence rates of ulcerative colitis (UC) during bDMARD non-exposure, adalimumab exposure, etanercept exposure, golimumab exposure, infliximab exposure, secukinumab exposure and ixekizumab exposure were 12.25, 9.69, 13.23, 9.97, 8.92, 14.98 and 0.00 per 10 000 PYs, respectively (Table 5). As the PYs of ixekizumab exposure was too short and no events occurred during this short exposure duration, ixekizumab exposure was excluded in the Cox models.

**Table 5.** Incidence rates of IBD

	Events	Patient-years	IR/10 000 patient-years (95% CI)
IBD			
Non-exposure	325	177 686.4	18.29 (16.30, 20.28)
Adalimumab	41	20 522.1	19.98 (13.87, 26.09)
Etanercept	22	9816.3	22.41 (13.06, 31.77)
Golimumab	17	9016.6	18.85 (9.90, 27.81)
Infliximab	14	8902.2	15.73 (7.49, 23.96)
Secukinumab	3	666.8	44.99 (−5.81, 95.79)
Ixekizumab	0	20.2	0.00 (0.00, 0.00)
CD			
Non-exposure	107	178 463.9	6.00 (4.86, 7.13)
Adalimumab	21	20 660.3	10.16 (5.82, 14.51)
Etanercept	9	9844. 1	9.14 (3.17, 15.11)
Golimumab	8	9078.2	8.81 (2.71, 14.92)
Infliximab	6	8979.0	6.68 (1.34, 12.03)
Secukinumab	2	670.1	29.85 (0.00, 71.15)
Ixekizumab	0	20.2	0.00 (0.00, 0.00)
UC			
Non-exposure	218	177 946.0	12.25 (10.63, 13.88)
Adalimumab	20	20 648.0	9.69 (5.44, 13.93)
Etanercept	13	9824.5	13.23 (6.04, 20.42)
Golimumab	9	9031.3	9.97 (3.46, 16.47)
Infliximab	8	8964.1	8.92 (2.74, 15.11)
Secukinumab	1	667.3	14.98 (−14.36, 44.33)
Ixekizumab	0	20.2	0.00 (0.00, 0.00)

CD: Crohn’s disease; IBD: inflammatory bowel disease; IR: incidence rate; UC: ulcerative colitis.

### Risk of incident IBD across different bDMARDs

Table 6 presents the HRs for incident IBD according to bDMARD exposure. Compared with bDMARD non-exposure, adalimumab exposure (aHR, 0.880; 95% CI: 0.632, 1.224), etanercept exposure (aHR, 1.001; 95% CI: 0.647, 1.547), golimumab exposure (aHR, 0.922; 95% CI: 0.564, 1.508), infliximab exposure (aHR, 0.692; 95% CI: 0.404, 1.186) and secukinumab exposure (aHR, 1.807; 95% CI: 0.573, 5.698) did not show significant difference in the risk of IBD. Similar results were observed when CD and UC were analysed separately. When adalimumab exposure was used as the comparator, etanercept exposure (aHR, 1.118; 95% CI: 0.665, 1.878), golimumab exposure (aHR, 0.932; 95% CI: 0.525, 1.657), infliximab exposure (aHR, 0.774; 95% CI: 0.421, 1.422) and secukinumab exposure (aHR, 2.061; 95% CI: 0.624, 6.814) showed no significant difference in risk of IBD. Similar results were observed when CD and UC were analysed separately. In the exploratory subgroup analyses, similar results were observed across all subgroups (Supplementary Fig. S4, available at *Rheumatology* online).

**Table 6.** Risk of incident IBD according to bDMARD exposure

	Univariable model		Multivariable model	
	Unadjusted HR (95% CI)	*P*	Adjusted HR^a^ (95% CI)	*P*
IBD				
bDMARD non-exposure *vs*				
Adalimumab	1.100 (0.795, 1.523)	0.564	0.880 (0.632, 1.224)	0.447
Etanercept	1.237 (0.803, 1.905)	0.335	1.001 (0.647, 1.547)	0.997
Golimumab	1.047 (0.642, 1.709)	0.853	0.922 (0.564, 1.508)	0.746
Infliximab	0.864 (0.506, 1.476)	0.593	0.692 (0.404, 1.186)	0.181
Secukinumab	2.485 (0.794, 7.784)	0.118	1.807 (0.573, 5.698)	0.312
Adalimumab *vs*				
Etanercept	1.125 (0.670, 1.888)	0.657	1.118 (0.665, 1.878)	0.674
Golimumab	0.928 (0.525, 1.642)	0.798	0.932 (0.525, 1.657)	0.811
Infliximab	0.792 (0.431, 1.454)	0.451	0.774 (0.421, 1.422)	0.409
Secukinumab	2.071 (0.632, 6.779)	0.229	2.061 (0.624, 6.814)	0.236
CD				
bDMARD non-exposure *vs*				
Adalimumab	1.683 (1.054, 2.688)	0.029	1.389 (0.860, 2.245)	0.179
Etanercept	1.546 (0.783, 3.054)	0.210	1.295 (0.651, 2.576)	0.461
Golimumab	1.350 (0.656, 2.775)	0.415	1.192 (0.578, 2.458)	0.635
Infliximab	1.123 (0.493, 2.556)	0.782	0.934 (0.408, 2.142)	0.873
Secukinumab	4.416 (1.081, 18.031)	0.039	3.345 (0.807, 13.867)	0.096
Adalimumab *vs*				
Etanercept	0.909 (0.416, 1.984)	0.810	0.897 (0.410, 1.959)	0.784
Golimumab	0.819 (0.361, 1.858)	0.633	0.814 (0.356, 1.861)	0.625
Infliximab	0.669 (0.270, 1.659)	0.385	0.655 (0.263, 1.628)	0.362
Secukinumab	2.596 (0.597, 11.288)	0.203	2.704 (0.612, 11.949)	0.190
UC				
bDMARD non-exposure *vs*				
Adalimumab	0.803 (0.508, 1.270)	0.349	0.632 (0.398, 1.006)	0.053
Etanercept	1.088 (0.621, 1.904)	0.769	0.865 (0.492, 1.521)	0.615
Golimumab	0.878 (0.449, 1.717)	0.704	0.772 (0.394, 1.512)	0.451
Infliximab	0.731 (0.361, 1.480)	0.384	0.573 (0.282, 1.165)	0.124
Secukinumab	1.340 (0.187, 9.608)	0.771	0.954 (0.132, 6.881)	0.963
Adalimumab *vs*				
Etanercept	1.361 (0.677, 2.736)	0.388	1.367 (0.679, 2.752)	0.382
Golimumab	1.051 (0.474, 2.328)	0.903	1.072 (0.481, 2.389)	0.864
Infliximab	0.918 (0.404, 2.087)	0.838	0.894 (0.393, 2.037)	0.790
Secukinumab	1.478 (0.195, 11.209)	0.706	1.406 (0.183, 10.779)	0.743

a Adjusted for age, sex, psoriasis, uveitis, methotrexate, sulfasalazine, glucocorticoids, non-selective NSAIDs and selective cyclooxygenase-2 inhibitors. bDMARD: biologic DMARD; CD: Crohn’s disease; IBD: inflammatory bowel disease; HR: hazard ratio; UC: ulcerative colitis.

## Discussion

Our real-world study describes the long-term SPSI (TB and IBD) in Korean patients with AS receiving biologic agents. Overall, exposure of IL-17is was associated with numerically lower incidence of TB and numerically higher incidence of IBD compared with TNFi exposure. When looking at individual biologic agents, compared with bDMARD non-exposure, adalimumab exposure, golimumab exposure and infliximab exposure were associated with a higher risk of TB; conversely, etanercept and secukinumab exposures showed no significant increase in the risk of TB. With etanercept exposure as a comparator, adalimumab exposure, golimumab exposure and infliximab exposure were associated with a higher risk of TB whereas secukinumab exposure was not associated with a higher risk of TB.

The results observed in this study were found to be similar to previously reported findings. In a study from Korea in patients (rheumatoid arthritis, AS, psoriatic arthritis and IBD) receiving TNFis, Jung *et al.* reported that the incidence of TB was higher in patients treated with infliximab (incidence rate ratio [IRR], 6.8; 95% CI: 3.74, 12.37) and adalimumab (IRR, 3.45; 95% CI: 1.82, 6.55) when compared with patients treated with etanercept as reference [28]. Another Korean study using the nationwide insurance claims database of the HIRA Service in patients with AS receiving TNFis reported that patients receiving infliximab showed a significantly higher IRR of TB than those receiving etanercept (IRR, 9.05; 95% CI: 1.10, 74.54) [29]. In a Brazilian study among rheumatic disease patients, patients receiving adalimumab presented a higher risk for TB compared with etanercept users (risk ratio [RR], 3.11; 95% CI: 1.16, 8.35) [30].

In a large, pooled cohort study from 28 clinical trials (19 trials in psoriasis, five trials in psoriatic arthritis and four trials in AS) of secukinumab involving 12 319 patients, no active cases of TB were reported as an AE for any indication [31]. A recent pooled analysis that combined data from 47 clinical trials of secukinumab, including 30 trials in psoriasis, nine in psoriatic arthritis, and eight in AS, which included a total of 15 644 patients, with an overall exposure of 27 765 PYs, reported that there were rare cases of mycobacterial infections (RR, 0.03/100 PYs). The study also examined post-marketing surveillance data, which encompassed a much larger sample size of 1 159 260 PYs. During this period, TB and latent TB infections (RR 0.02 and 0.008/100 PYs, respectively) with secukinumab treatment were uncommon [32].

A study reviewing the evidence for TB risk in patients with autoimmune rheumatic diseases treated with JAKis or bDMARDs, other than TNFis, based on randomized controlled trials and long-term extension studies, reported that the risk of TB was generally lower with the use of most of the non-TNFi agents, when compared with TNFis. The study also reported that the risk of *de novo* TB infection or reactivation of latent TB was low with apremilast, ustekinumab, secukinumab and rituximab treatment [13].

Evidence from real world data and clinical trials suggests that patients treated with TNFis have an increased risk of new TB infection or latent TB infection reactivation [33–36]. In a recent retrospective, observational, multinational study in patients with psoriasis and latent TB infection, IL-17 or IL-23 inhibitors did not appear to have an increased risk of TB reactivation and were recommended to be preferred over TNFis especially when TB reactivation is a concern [35].

In the subgroup analysis, golimumab exposure was associated with a particularly higher risk of TB in patients with CKD. Based on this finding, other biologic agents could be preferable in patients with CKD in terms of TB risk. However, further studies are needed to reveal the mechanism underlying this finding.

The results for IBD from our study highlight that when compared with bDMARD non-exposure, none of the biologic agents showed significant difference in the risk of IBD. Similarly, when adalimumab exposure was used as the comparator, other biologics showed no significant difference in the risk of IBD. The association of TNFis and IBD has been studied previously. The occurrence of new onset and flare of IBD is found to be rare among patients with AS who are undergoing anti-TNF therapy [37]. TNFis are used for the management of IBD and have resulted in notable advancements in patient outcomes [38]. A study evaluating the incidence of IBD AEs across 75 adalimumab trials reported that the incidence of IBD was low across diseases. The rate of IBD events occurring over 1 year of adalimumab exposure was 0.5 (95% CI: 0.1, 0.6)/100 PYs for AS [39]. In contrast, a large, real-world study from the USA reported that patients with AS who were treated with TNFis had higher incidence rates of newly diagnosed IBD (HR, 2.0; including CD [HR, 2.45] and UC [HR, 1.65]) compared with patients not treated with TNFis [40]. The association between the usage of anti-TNF treatments and the development of IBD has been described as a paradoxical effect of these therapies as a possible explanation [41].

Similarly, the association of IL-17is and IBD has also been extensively studied. A large safety analysis (*n* = 7355; cumulative exposure = 162 260.9) across 21 clinical trials reported that the occurrence of IBD events was infrequent in patients undergoing treatment with secukinumab. Furthermore, the observed EAIR of IBD did not increase over time [42]. In addition, in a comprehensive meta-analysis, which included >19 000 patients with an exposure of over 6 years undergoing treatment for psoriasis, psoriatic arthritis, AS, or rheumatoid arthritis with secukinumab, ixekizumab or brodalumab, no evidence was found to suggest an elevated risk of developing IBD among these patients [43].

A study using the US FDA Adverse Event Reporting System database reported that IL-17is treatment is associated with exacerbation and new onset of IBD and colitis [44]. The incidence of CD with secukinumab was reported to be higher than UC in our study. A previous study has reported similar results. In an integrated analysis of pooled data from clinical trials and post-marketing surveillance of secukinumab over a 5-year period, the incidence of CD was reported to be 0.4%, while that of UC was 0.2% in patients with AS [45]. Secukinumab may be associated with the worsening of CD; while the underlying cause and pathophysiological mechanisms have not been fully outlined, blocking IL-17A might interfere with its protective role in the intestine, which could be a possible explanation [46]. Therefore, it is crucial to gather a comprehensive patient history prior to initiating treatment with IL-17is and to actively monitor gastrointestinal symptoms and intestinal inflammatory biomarkers throughout the course of treatment. These measures are essential for ensuring the safe and appropriate use of these biologics [44].

This study has certain limitations that warrant acknowledgement. While we meticulously adjusted for covariates influencing the selection of bDMARDs within the multivariable model, it is imperative to recognize that this was a retrospective study, and the potential for confounding by indication remains. Additionally, given the nature of utilizing a claims database, intricate disease characteristics of AS, such as the presence of peripheral symptoms of spondyloarthritis, were not accessible. In addition, the diagnosis of IBD in our study was based on ICD-10 codes and was not a histologically confirmed diagnosis. Furthermore, the differentiation between pre-existing IBD and new-onset IBD was not feasible. Moreover, the duration of exposure to IL-17is was relatively brief. Lastly, the study exclusively focused on Korean patients, thus constraining the generalizability of our findings to other ethnic groups. Consequently, there is a need for further research encompassing prolonged IL-17i exposure durations and diverse ethnic populations to enhance the comprehensiveness of our understanding.

In conclusion, this study shows that the EAIRs of TB and IBD with the use of IL-17is in patients with AS were within the anticipated low range. IL-17is showed numerically lower incidence of TB, and numerically higher incidence of IBD compared with TNFis. When compared with bDMARD non-exposure, the risk of TB was significantly higher with TNFis (adalimumab, golimumab and infliximab). Secukinumab was not associated with the risk of incident TB when compared with bDMARD non-exposure, highlighting that IL-17is did not increase the EAIR for TB in patients with AS. In terms of IBD, neither TNFis nor IL-17is showed increased risk when compared with bDMARD non-exposure. Overall, these outcomes confirm the favourable safety profile of secukinumab, without increasing risk of TB or IBD, in managing patients with AS. These data are reassuring and provide valuable information, which could inform treating physicians.

## Supplementary Material

keaf038_Supplementary_Data

## Data Availability

The datasets generated and/or analysed during the current study are not publicly available. Novartis is committed to sharing access to patient-level data and supporting clinical documents from eligible studies with qualified external researchers. These requests are reviewed and approved on the basis of scientific merit. All data provided are anonymized to respect the privacy of patients who have participated in the trial in line with applicable laws and regulations. The data may be requested from the corresponding author of the manuscript.
