# Supplementary material for: Incidence rates of tuberculosis and inflammatory bowel disease in patients with ankylosing spondylitis treated with biologics in Korea
Source: Rheumatology (Oxford). 2025 Jan 24;64(6):3518–25. doi: 10.1093/rheumatology/keaf038 (PMC12107047; doi:10.1093/rheumatology/keaf038)
Supplement: keaf038_Supplementary_Data [file keaf038_supplementary_data.docx]

**Incidence Rates of Tuberculosis and Inflammatory Bowel Disease in Patients with Ankylosing Spondylitis Treated with Biologics in Korea**

Oh Chan Kwon^1*^MD, PhD, Hye Sun Lee^2*^ PhD, Juyeon Yang^2^ MS, Thomas Paul^3^ MD, Hyerim Jin^3^ PhD, Youkyung Lee^3^ MD, MSc, Min-Chan Park^1†^ MD, PhD

*Oh Chan Kwon and Hye Sun Lee contributed equally as co-first authors.

**^1^**Division of Rheumatology, Department of Internal Medicine, Yonsei University College of Medicine, Seoul, South Korea; ^2^Biostatistics Collaboration Unit, Yonsei University College of Medicine, Seoul, South Korea; ^3^Novartis Korea Ltd. Seoul, South Korea
**^†^Correspondence to:** Min-Chan Park, Yonsei University College of Medicine, Gangnam Severance Hospital, 211 Eonjuro, Gangnam-gu, Seoul 06273, Korea.

**Supplementary Figure S1.** Summary of the TB study cohort and exposures.


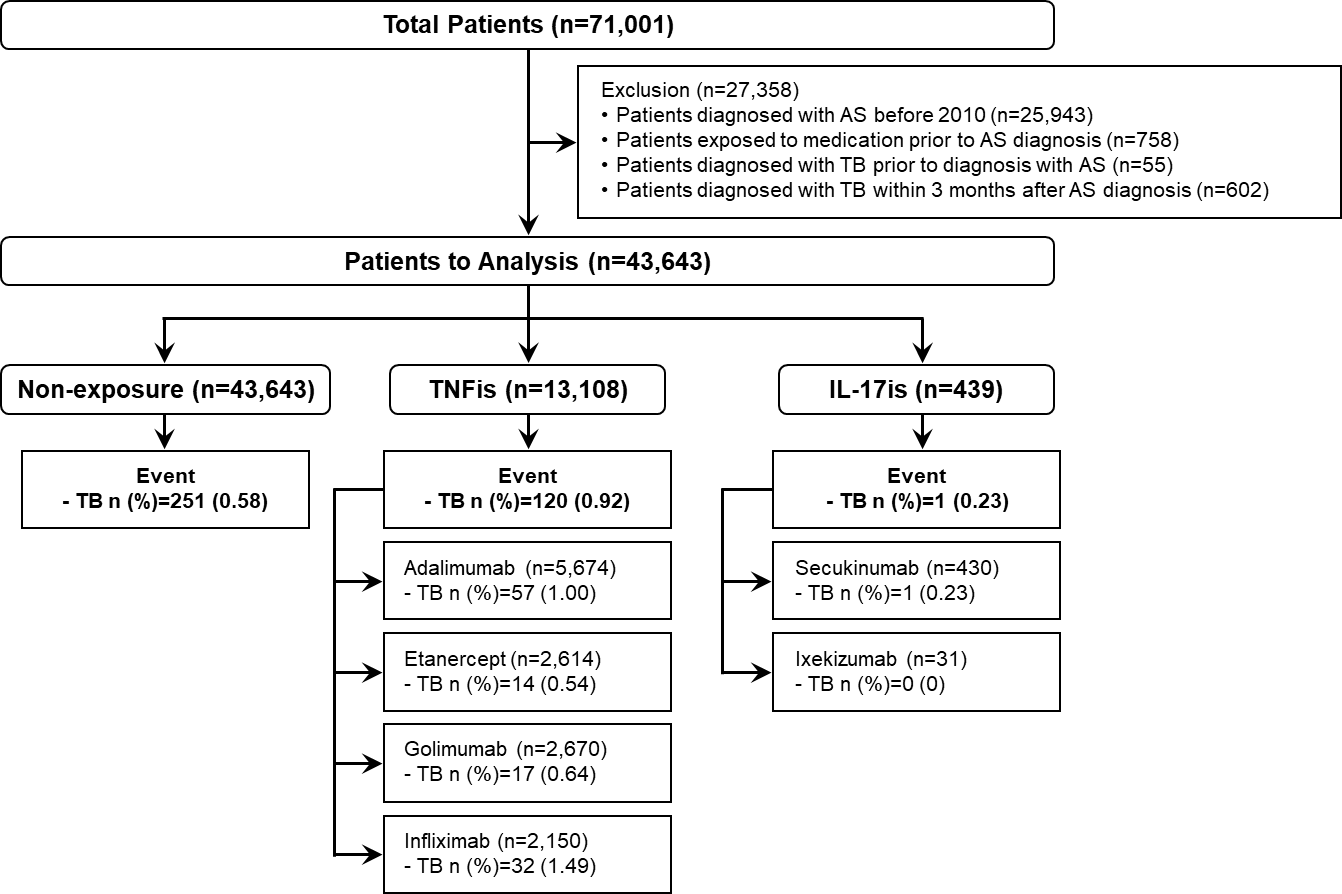


AS, ankylosing spondylitis; IL-17is, interleukin-17 inhibitors; n, number of patients;
TB, tuberculosis; TNFis, tumor necrosis factor inhibitors.

**Supplementary Figure S2.** Summary of the IBD study cohort and exposures.


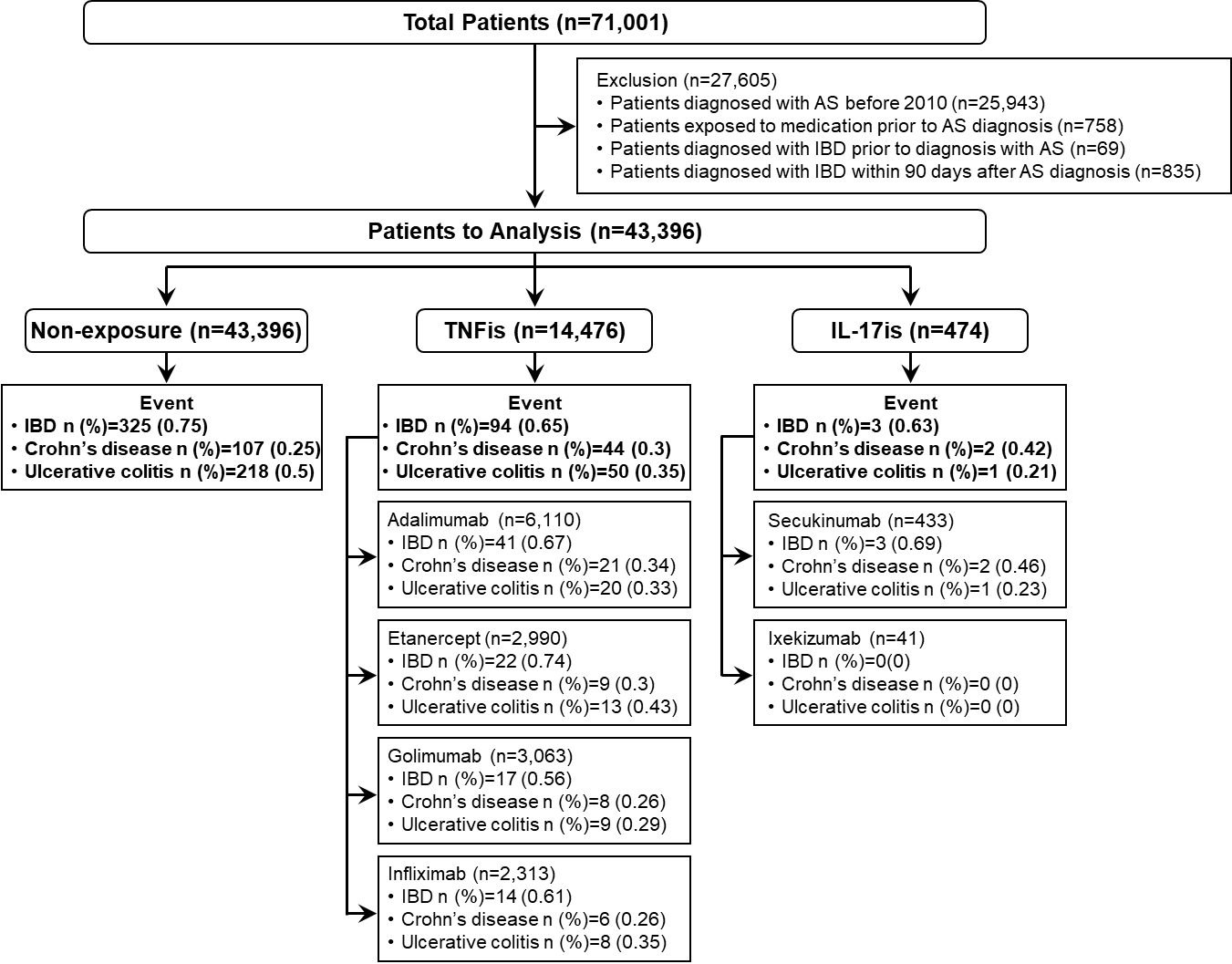


AS, ankylosing spondylitis; IBD, inflammatory bowel disease; IL-17is, interleukin-17 inhibitors; n, number of patients; TNFis, tumor necrosis factor inhibitors.

**Supplementary Figure S3.** **Subgroup analyses of TB**


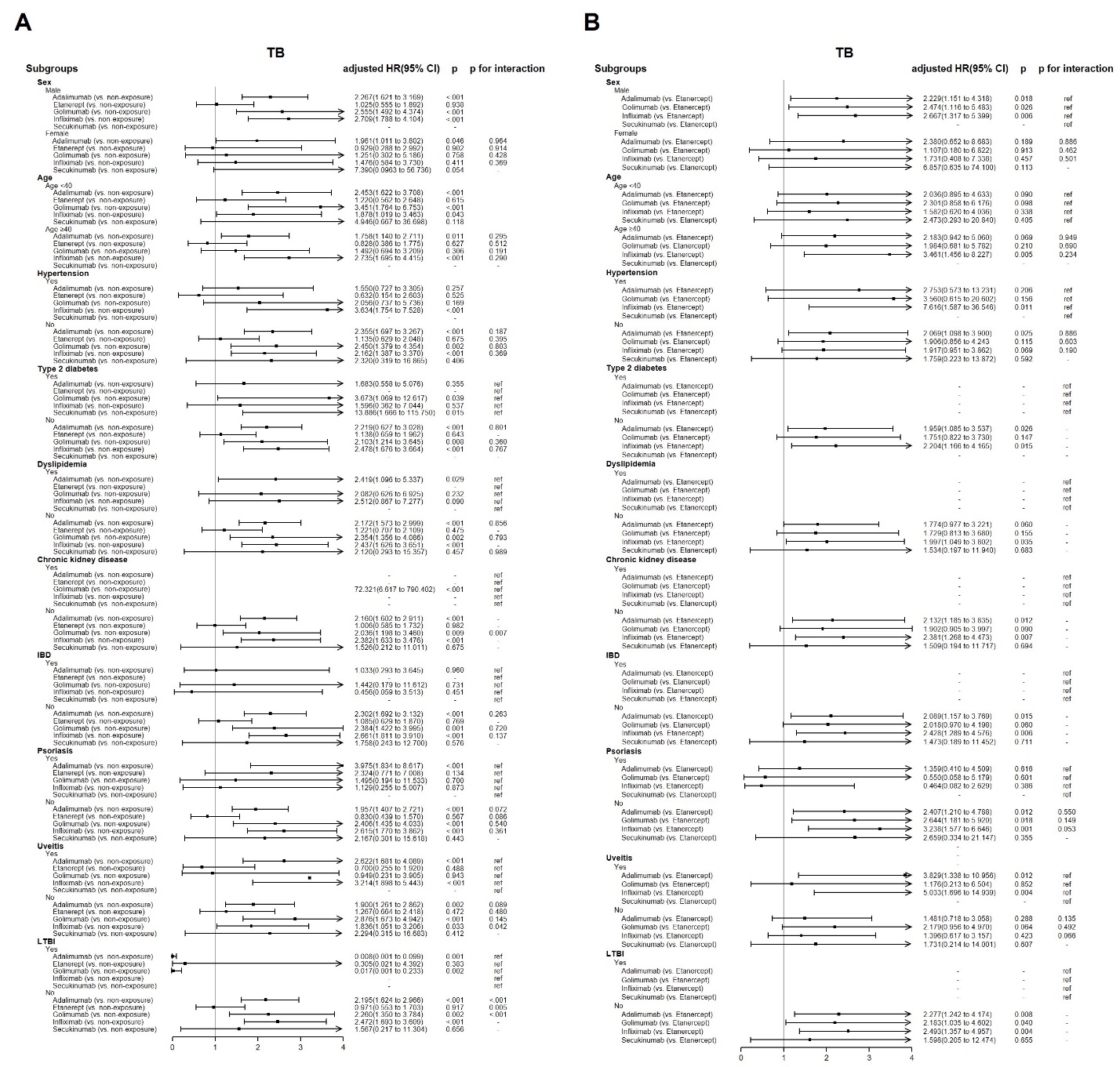


**Supplementary Figure S4.** **Subgroup analyses of IBD**


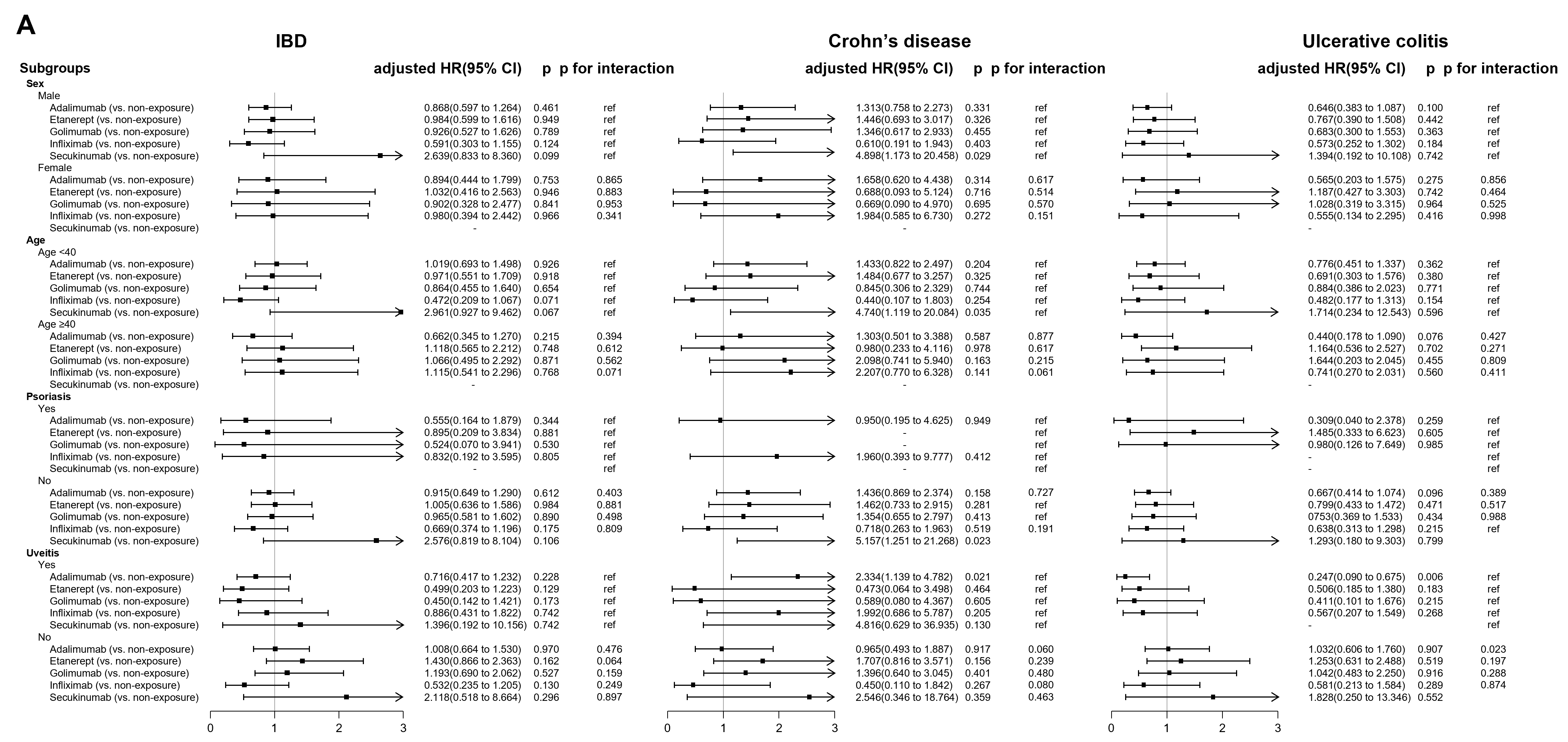


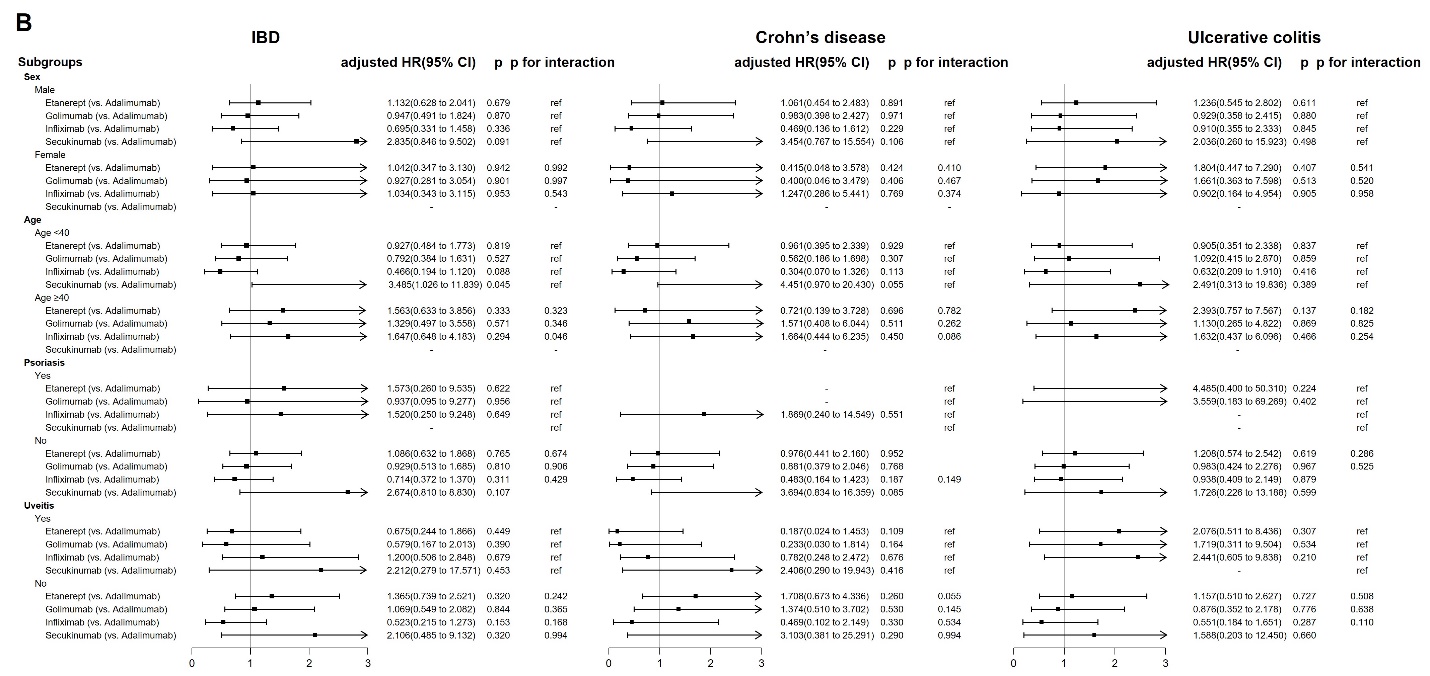


IBD, inflammatory bowel disease.
